# 

**Published:** 1915-09

**Authors:** 


### CONTENTS, SEPTEMBER, 1915—VOLUME XXXI, NO. 3.

ORIGINAL ARTICLES.

Skiagraphic Study of Thorax, Thoracic Wall and Thoracic Viscera .... 87

A Tribute to Dr. M. B. Grace .... 103

DEPARTMENT OF EUGENICS.

Dr. Kellogg on Aristocracy .... 107

Heredity and Environment. By Edwin G. Franklin .... 108

EDITORIAL DEPARTMENT.

The Family Physician. By Dr. T. D. Crothers .... 113

War Babies. By Mrs. Bessie Drsydale .... 116

Items of General Interest .... 119

Personal News Items .... 122

Helpful Hints for the Busy Doctors .... 128

